# Structure and Function of the *Escherichia coli* Tol-Pal Stator Protein TolR*

**DOI:** 10.1074/jbc.M115.671586

**Published:** 2015-09-09

**Authors:** Justyna A. Wojdyla, Erin Cutts, Renata Kaminska, Grigorios Papadakos, Jonathan T. S. Hopper, Phillip J. Stansfeld, David Staunton, Carol V. Robinson, Colin Kleanthous

**Affiliations:** From the ‡ Department of Biochemistry, University of Oxford, South Parks Road, Oxford OX1 3QU and; the § Department of Chemistry, University of Oxford, Mansfield Road, Oxford OX1 3TA, United Kingdom

**Keywords:** bacteria, crystal structure, dimerization, Escherichia coli (E. coli), membrane protein, TolR, domain, periplasm, strand-swapped

## Abstract

TolR is a 15-kDa inner membrane protein subunit of the Tol-Pal complex in Gram-negative bacteria, and its function is poorly understood. Tol-Pal is recruited to cell division sites where it is involved in maintaining the integrity of the outer membrane. TolR is related to MotB, the peptidoglycan (PG)-binding stator protein from the flagellum, suggesting it might serve a similar role in Tol-Pal. The only structure thus far reported for TolR is of the periplasmic domain from *Haemophilus influenzae* in which N- and C-terminal residues had been deleted (TolR(62–133), *Escherichia coli* numbering). *H. influenzae* TolR(62–133) is a symmetrical dimer with a large deep cleft at the dimer interface. Here, we present the 1.7-Å crystal structure of the intact periplasmic domain of *E. coli* TolR (TolR(36–142)). *E. coli* TolR(36–142) is also dimeric, but the architecture of the dimer is radically different from that of TolR(62–133) due to the intertwining of its N and C termini. TolR monomers are rotated ∼180° relative to each other as a result of this strand swapping, obliterating the putative PG-binding groove seen in TolR(62–133). We found that removal of the strand-swapped regions (TolR(60–133)) exposes cryptic PG binding activity that is absent in the full-length domain. We conclude that to function as a stator in the Tol-Pal complex dimeric TolR must undergo large scale structural remodeling reminiscent of that proposed for MotB, where the N- and C-terminal sequences unfold in order for the protein to both reach and bind the PG layer ∼90 Å away from the inner membrane.

## Teaser

**Background:** TolR forms part of the proton-motive force (pmf)-linked stator of the Tol-Pal complex in bacteria.

**Results:** Structural and biophysical analysis shows the periplasmic domain of TolR to be a strand-swapped dimer.

**Conclusion:** Strand swapping blocks peptidoglycan binding by TolR.

**Significance:** Pmf-linked stator proteins must disassemble strand-swapped dimer structures to traverse the periplasm and bind peptidoglycan.

## Introduction

The architecture of the Gram-negative cell envelope poses a significant problem to bacteria for processes requiring access to an energy source, such as building of the outer membrane (OM),^4^ expulsion of xenobiotics, and motility. Bacteria overcome this problem by coupling these processes to the hydrolysis of ATP in the cytoplasm or the proton motive force (pmf) across the inner membrane (IM). Such coupling requires protein machines that transduce the expended energy into work across the cell envelope, as epitomized by the bacterial flagellum. Proton movement through the stator proteins MotA and MotB in the IM are coupled to interactions with PG and rotor proteins driving rotation of the flagellum (1). Proteins related to MotA and MotB are also involved in coupling the pmf to other energy-dependent processes in the cell envelope. For example, ExbB/ExbD coordinate with the trans-periplasmic TonB protein to drive nutrient influx through numerous OM transporters (2), and AglR/AglS drives gliding motility along surfaces in *Myxococcus xanthus* (3). Here, we investigate the MotB/ExbD/AglS paralogue TolR from *Escherichia coli* K12 and its potential role as the stator for the Tol-Pal complex.

The Tol-Pal system is widely distributed in Gram-negative bacteria but notably absent in intracellular parasites such as Chlamydiae (4). The function of the complex, which is a virulence factor in pathogens such as *Salmonella enterica* (5), *Pseudomonas aeruginosa* (6), and *Erwinia chrysanthemi* (7), is currently unknown but is thought to be linked to OM integrity. *tol-pal* mutations or deletions disrupt the OM leading to increased membrane blebbing, release of periplasmic contents, and hypersensitivity toward detergents, antibiotics, and bile salts (8). The Tol-Pal assembly is recruited to cell division sites where it is involved in invagination of the OM, which is consistent with the cell-chaining phenotype and aberrant positioning of septation sites often seen in *tol-pal* mutants (9). Tol-Pal has also been implicated in polar localization of chemoreceptors in the IM and lipopolysaccharide O-antigen polymerization (10, 11). Finally, Tol-Pal is parasitized by some filamentous bacteriophages and group A colicins where it is exploited for the translocation of DNA or cytotoxic proteins, respectively, into the cell (8, 12).

The Tol-Pal system has at its core five proteins, the genes of which are expressed constitutively from two operons and are up-regulated through the Rcs cell envelope stress pathway (13). TolQ, TolR, and TolA are IM proteins that are coupled to the pmf through a conserved proton-conducting path, which is also found in MotA/MotB, ExbB/ExbD, and AglR/AglS. The lipoprotein Pal and periplasmic protein TolB are unique to the Tol-Pal system and form a complex at the OM (14). The pmf drives structural transitions in TolA, which extends through the periplasm (15). TolA forms a weak interaction with TolB and possibly Pal, although the latter remains controversial (16, 17). Pal also interacts with PG, an interaction that is mutually exclusive of its interaction with TolB (18).

TolQ, TolR, and TolA form a complex in the IM, where the stoichiometry has been estimated as 4–6:2:1 (19). TolQ contains three transmembrane (TM) helices, and both TolR and TolA are embedded in the IM through single TM helices. A model for how the TM helices of the three components associate has been proposed based on allelic-specific suppressor mutations, accessibility of unique cysteines to chemical modification, and disulfide bond cross-linking (20–22). From these studies it has been proposed that the C-terminal two TM helices of TolQ contact TolR to form part of the proton-conducting pore. Positioned within this pore is a highly conserved aspartate residue (Asp-23) in TolR, which is thought to be one of the sites of protonation and has an equivalent site in the TM helices of MotB and ExbB (23). The first TM helix of TolQ contacts a conserved SHLS motif in the TM helix of TolA that also forms part of the pmf-coupling mechanism. The proton gradient influences the orientations of the TolQ/TolR TM helices, and it has been proposed that they rotate relative to each other thereby providing the driving force for the structural transitions in the Tol-Pal assembly (22).

This work focuses on the periplasmic domain of TolR from *E. coli*. By analogy with the stator protein MotB, this domain is postulated to act as a plug for the proton-conducting pore of the Tol-Pal complex (21). Such a role requires TolR to exist in different conformational states as follows: a closed state blocking the pore and an open state allowing protons to pass through the pore. Consistent with this hypothesis, cysteine accessibility studies *in vivo* have shown that the C-terminal 27 amino acids of the periplasmic domain of TolR exist in different conformational states that are influenced by the pmf and are predicted to constitute part of the plug (21). Another key attribute of the MotB stator is its ability to bind the PG layer. No such interaction has been demonstrated for TolR. Moreover, all previous studies localize the TolR periplasmic domain far from the PG layer, including the C-terminal 27 amino acids which are thought to interact with the IM (21, 24). Here, we reconcile these contradictory observations through structural, biochemical, and biophysical studies on the periplasmic domain of TolR from which we propose a mechanism for the structural transition of this stator protein in the bacterial periplasm.

## Experimental Procedures

### 

#### 

##### Protein Expression and Purification

DNA sequence encoding different versions of the *E. coli* soluble domain or full-length TolR was cloned into the expression vector pETM-11 (European Molecular Biology Laboratory) with the N-terminal His_6_ tag followed by tobacco etch virus protease cleavage site. Cleavage of protease leaves additional three residues GAM at the N terminus of the protein. TolR constructs, pJAW32 encoding TolR(1–142) (expected molecular mass of the monomer after removal of the tag is 15,511 Da), pJAW21 encoding TolR(36–142) (11,695.4 Da), pJAW23 encoding TolR(36–133) (10,768.2 Da), pREN24 encoding TolR(60–133) (8314.6 Da), and pREN42 encoding TolR(64–142) (8818.3 Da), were expressed in *E. coli* strain BL21 DE3.

##### Soluble Domains of TolR

Cultures were grown in LB at 37 °C until the *A*_600_ reached 0.8, induced with 1 mm isopropyl 1-thio-β-d-galactopyranoside, and left shaking overnight at 20 °C. Harvested cells were stored at −20 °C. Pellets from 5-liter cell cultures were resuspended in 50 ml of lysis buffer (50 mm Tris, pH 7.5, 500 mm NaCl), lysed by sonication, and centrifuged at 13,000 rpm. The filtered supernatant was loaded onto a 5-ml HisTrap HP column (GE Healthcare) equilibrated with lysis buffer, and protein was eluted with a linear gradient of imidazole (50 mm Tris, pH 7.5, 500 mm NaCl, 500 mm imidazole). Fractions containing TolR were pooled and dialyzed overnight against 50 mm Tris, pH 7.5, 300 mm NaCl. To remove the His_6_ tag, the isolated protein was incubated with tobacco etch virus protease (produced in-house) at a 50:1 mg ratio for 4 h at room temperature. Uncleaved material and His_6_-tagged tobacco etch virus protease were removed by incubating the mixture with 5 ml of nickel-nitrilotriacetic acid beads (Qiagen). Cleaved protein was then loaded onto a Superdex75 26/60 gel filtration column (GE Healthcare) equilibrated in 50 mm Tris, pH 7.5, and 500 mm NaCl. Eluted fractions were analyzed for purity by Coomassie-stained 16% SDS-polyacrylamide gel. Fractions containing pure TolR were pooled and stored at −20 °C. Because of the tagging/cleavage strategy used in this study, all TolR proteins retained three residues from the tag (Gly-Ala-Met) at the N terminus.

##### Full-length TolR(1–142)

Bacteria were grown in TB at 37 °C until *A*_600_ reached 0.8, cooled on ice for 5 min, induced with 1 mm isopropyl 1-thio-β-d-galactopyranoside, and left shaking overnight at 20 °C. Harvested cells from a 5-liter culture were incubated for 1 h at 4 °C and mixed with 120 ml of lysis buffer (50 mm Tris, pH 7.5, 500 mm NaCl) containing 40 mg of chicken egg white lysozyme (Sigma) and 6 μl of benzonase (Sigma). After lysis by sonication, the cells were centrifuged at 13,000 rpm. Membranes were separated by ultracentrifugation of the supernatant at 230,000 × *g,* then resuspended in 40 ml of lysis buffer containing 40 mm LDAO, and incubated for 1 h at 4 °C. Solubilized material was isolated by ultracentrifugation at 200,000 × *g* and loaded onto a 5-ml HisTrap HP column equilibrated with lysis buffer containing 4.4 mm LDAO. Elution was performed with a linear gradient of imidazole (50 mm Tris, pH 7.5, 500 mm NaCl, 500 mm imidazole, 4.4 mm LDAO). Eluted protein was subsequently loaded onto a Superdex75 16/60 gel filtration column (GE Healthcare) equilibrated in 50 mm Tris, pH 7.5, 300 mm NaCl, 4.4 mm LDAO. The protein was deemed pure by a Coomassie-stained 16% SDS-polyacrylamide gel.

##### Size Exclusion Chromatography Multiangle Laser Light Scattering (SEC-MALLS)

100-μl samples of TolR constructs at various protein concentrations were loaded onto a Superdex75 10/300 (GE Healthcare) gel filtration column connected to a Shimadzu HPLC and equilibrated in 50 mm Tris, pH 7.5, 150 mm NaCl at a flow rate of 0.5 ml/min. Elution was monitored with a DAWN HELEOS-II 8-angle light scattering detector (Wyatt Technology), a SPD-20A UV/VIS detector (Shimadzu), and an OPTILab rEX refractive index monitor (Wyatt Technology). Data were analyzed with the program Astra 6 (Wyatt Technology), and molar masses of tested constructs were calculated.

##### Analytical Ultracentrifugation

Sedimentation velocity and equilibrium experiments were performed in a ProteomeLab XL-I analytical ultracentrifuge (Beckman Coulter) using an An-60 Ti rotor at 20 °C. Detection was performed with Rayleigh interference optics. In sedimentation equilibrium experiments, TolR(36–142) was centrifuged at 15,000, 20,000, 25,000, 30,000, and 35,000 rpm for 20 h at each speed in 50 mm Tris, pH 7.5, 150 mm NaCl and at 20 and 200 μm concentration. Collected data were analyzed with Optima XL Data Analysis Software (Beckman Coulter). In sedimentation velocity experiments, TolR(36–142) at 100 μm was centrifuged at 40,000 rpm. The program SEDFIT (25) was used to calculate continuous distribution of sedimentation coefficient, *c*(*M*). Partial specific volume and buffer density were calculated with the program SEDNTERP (26).

##### Native State Electrospray Ionization Mass Spectrometry (ESI-MS)

Protein aliquots were desalted using Biospin-6 (Bio-Rad) columns equilibrated with 100 mm ammonium acetate. For full-length TolR (TolR(1–142)), buffers were supplemented with 0.05% LDAO. Approximately 2–3 μl of desalted sample (TolR(33–142) and TolR(1–142)) were loaded into gold-coated silica nanospray capillaries, prepared in-house using a procedure described previously. Capillaries were mounted to a static spray block of a quadrupole time-of-flight mass spectrometer, modified for high mass transmission, and spray was induced by applying between 1600 and 1800 V to the capillary. Pressure was maintained at ∼7 × 10^−3^ mbars in the source region of the instrument, which was necessary to improve transmission of protein complexes. TolR(1–142) was liberated from LDAO micelles in the collision cell of the mass spectrometer by accelerating detergent-protein complexes via a potential difference of 100 V into argon gas maintained at ∼0.2 MPa.

##### Crystallization and Structural Determination

Crystallization trials of TolR(36–142) at 38.5 mg/ml were performed using the hanging drop vapor diffusion method. Crystals were obtained in 0.1 m Hepes buffer, pH 7.5, 1.0 m sodium phosphate, and 0.8 m potassium phosphate. A single crystal was transferred into 3.0 m sodium malonate, pH 7.5, for cryoprotection and flash-frozen in liquid nitrogen. Single wavelength x-ray diffraction data containing 2000 images were collected from a single crystal at 100 K at the Diamond Light Source i04-1 beamline using the Pilatus 2 M detector. Crystal-to-detector distance was kept at 174.8 mm with an oscillation range of 0.2°. The crystal belonged to space group *P*6_5_22 with unit cell dimensions *a* = 51.7 Å, *b* = 51.7 Å, and *c* = 155.0 Å. Recorder images were processed with XDS (27), the reflection intensities were processed with COMBAT and scaled with SCALA (28, 29) from the CCP4 program suite (29). The structure was determined by molecular replacement using the program Phaser (30) from the Phenix software (31). An ensemble consisting of *Haemophilus influenzae* TolR (PDB code 2JWK) and *E. coli* ExbD periplasmic domain (PDB code 2PFU) structures served as search models. The solution was refined with phenix.refine, and a more complete model was obtained with phenix.autobuild. Further refinement was carried out using the program REFMAC5 (32). The structure was visualized and rebuilt into electron density using the program Coot (33). The stereochemistry of the model was evaluated with the program MolProbity (34). Data collection and refinement statistics are shown in Table 2. Atomic coordinates and structural amplitudes have been deposited with the Protein Data Bank (PDB code 5BY4).

##### TolR Peptidoglycan Binding Assay

Intact *E. coli* sacculi were isolated from D456 cells (35), according to previously described procedures (36). The preparation was quantified by digestion of NAG-NAM disaccharides using the muramidase mutanolysin (Sigma) whose enzymatic activity was in turn defined using the *Enterococcus faecalis* cell wall substrate and assay protocol from Sigma. ∼20 μmol from a 400 mm suspension was collected by ultracentrifugation using a Beckman TLA-100 rotor at 90,000 rpm at 25 °C for 30 min. The murein pellet was resuspended in 50 μl containing 5 nmol of TolR construct (57 μg of TolR24 and 73 μg of TolR21) in 10 mm Tris maleate, 10 mm MgCl_2_, 50 mm NaCl, pH 6.8 (buffer A). Control samples of TolR constructs without murein were also prepared. The samples were incubated at room temperature for 30 min and then centrifuged as above (binding step). The murein pellet was resuspended in 200 μl of buffer A and recovered by centrifugation. This wash step was repeated three times, and the final pellet was resuspended in 2% SDS and stirred for 1 h at room temperature. The supernatant of the binding step of the third wash step and the resuspended pellet were analyzed by 16% SDS-PAGE containing 8 m urea in the running gel. The His_6_ tag of the TolR constructs was detected using an anti-His HRP-conjugated antibody (Sigma) after blotting of the proteins onto a Hybond nitrocellulose membrane (GE Healthcare).

##### Molecular Dynamics (MD) Simulations

The model of TM-TolR(36–142) (TolR with the TM helix residues 15–142) was created using MODELER (37). Residues Ile-38–Pro-141 were as described by the structural coordinates, and residues Pro-20–Pro-37 were modeled as helical, based on secondary structure prediction servers Jpred (38) and PSIPRED (39), and all other residues were modeled as loops. A dimeric model was then made from two identical monomers mapped onto the x-ray structural coordinates. The TM-TolR(36–142) model was converted to coarse grain, with secondary structure preserved with elastic network restraints and embedded into an *E. coli* model of a bilayer consisting of phosphatidylglycerol and phosphatidylethanolamine at a 1:4 ratio (40, 41). Simulations were performed for 100 ns with the protein position restrained and then for an additional 100 ns without positional restraints. The coarse grain system was converted to the united atom force field GROMOS96 53a6 (42) and spc216 water model (43). All molecular dynamics simulations were run for 100 ns, in triplicate using GROMACS version 4.6. Energy minimization was performed to a tolerance of 100 kJ mol^−1^ nm^−1^, and a restrained run was performed for at least 200 ps prior to a 100-ns simulation. In all cases, a time step of 2 fs was used, with frames recorded every 10 ps. Pressure was maintained at 1 bar using the Parrinello-Rahman barostat (44), and temperature was maintained at 323 K using the V-rescale thermostat (45). Simulations of the crystal structure without modeled transmembrane region were performed for comparison and were also run for 100 ns with the GROMOS53a1 force field but with temperature maintained at 310 K. Secondary structural analysis was performed with GROMACS tools and DSSP (46, 47), electrostatic analysis with PDB code 2PQR (48, 49) and APBS (50), and simulation analysis with MDAnalysis (51). Molecular graphics were created using PyMOL (52).

## Results

### 

#### 

##### N- and C-terminal Sequences of the Periplasmic Domain Confer Stability to the E. coli TolR Dimer

**FIGURE 1.**
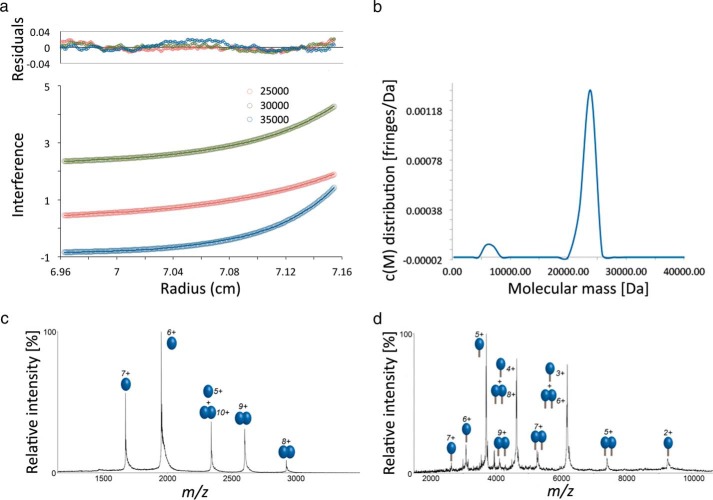
**Intact *E. coli* TolR is a dimer *in vitro*.** *a*, analytical ultracentrifugation sedimentation equilibrium analysis of the periplasmic domain of TolR, TolR(36–142) (20 μm), at three different centrifugation speeds. Also shown are the residuals to fits of the experimental data to a self-association model. *b,* sedimentation velocity *c*(*M*) distribution shows that TolR(36–142) is predominantly dimeric. *c* and *d,* native state ESI-MS of TolR(36–142) (2 μm) and intact TM-TolR(1–142) (30 μm), respectively (see “Experimental Procedures” for details).

The solution structure of the periplasmic domain of TolR from *H. influenzae* has been reported previously by Parsons *et al.* (24) using a combination of NMR spectroscopy and small angle x-ray scattering data. TolR is a compact dimer that has a deep groove at the dimer interface formed by β-sheets from each subunit. To obtain protein samples amenable to structural determination, Parsons *et al.* (24) truncated the periplasmic domain at both its N and C terminus, removing 29 amino acids in total. These regions of the protein are, however, implicated in function, especially the C-terminal nine residues that are involved in the pmf-driven structural transition (21). In this work, we investigated the impact of these sequences on the structure and function of *E. coli* TolR, which is 63% identical to *H. influenzae* TolR and three residues longer. Initially, we expressed and purified the entire periplasmic domain of *E. coli* TolR (TolR(36–142)) as well as intact TolR (TolR(1–142)), containing the transmembrane (TM) helix and a short N-terminal cytoplasmic region. The oligomeric structure of TolR(36–142) was that of a dimer in solution, as deduced by analytical ultracentrifugation experiments (Fig. 1, *a* and *b*). Native state electrospray ionization mass spectrometry (ESI-MS) confirmed the dimeric structure of the protein but also indicated the presence of monomer in approximately equal measure, and the high preponderance of monomer is likely the consequence of gas phase-induced dissociation (Fig. 1*c*). The same phenomenon was observed for TolR(1–142) suggesting the presence of TolR transmembrane and cytoplasmic sequences confer little additional stability on the dimer (Fig. 1*d*).

**FIGURE 2.**
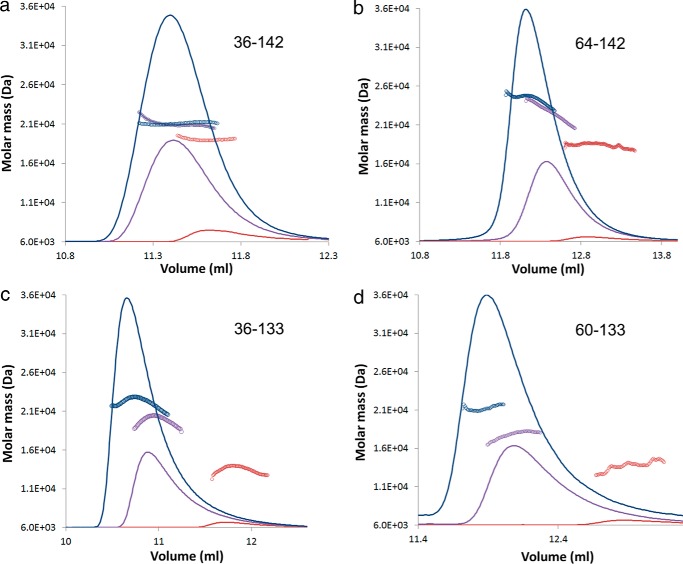
**Deletion of N- and C-terminal sequences from the periplasmic domain of TolR compromise dimer stability.** SEC-MALLS analysis of TolR(36–142) (*a*), TolR(64–142) (*b*) in which the N-terminal linker sequence to the TM was deleted, TolR(36–133) (*c*) in which C-terminal sequences were deleted and TolR(60–133) (*d*) in which both were deleted. All protein samples were analyzed at three protein concentrations: 500 μm (*blue symbols*), 200 μm (*purple*), and 20 μm (*red*).

We next analyzed the impact of truncating the termini of the periplasmic domain of *E. coli* TolR on its oligomeric stability. Although the study of Parsons *et al.* (24) on *H. influenzae* TolR established the protein was dimeric after truncation of its termini (24), their experiments were conducted at a high protein concentration where any change in dimer stability would not be evident. We therefore analyzed the oligomeric status of our TolR constructs over a range of protein concentrations (20, 200 or 500 μm) by SEC-MALLS (Fig. 2 and Table 1). The mass of the intact periplasmic domain (TolR(36–142)) remained largely unchanged over this range (Fig. 2*a*). Deleting 28 residues from the N terminus of TolR (TolR(64–142)) or 9 residues from the C terminus (TolR(36–133)) had a substantial effect on the stability of the dimer in SEC-MALLS experiments, with significant dissociation observed for either deletion at the lowest protein concentration (Fig. 2, *b* and *c*). Deleting the C-terminal 9 residues of the protein had the biggest impact on the stability of the dimer in solution, with the protein being essentially monomeric at low micromolar concentrations. Combining the deletions (TolR(60–133)) in a construct similar to that used by Parsons *et al.* (24) for the *H. influenzae* structural determination of TolR produced an even more destabilized dimer where subunit dissociation was observed at 200 μm (Fig. 2*d*). We conclude that the N and C termini of the periplasmic domain of *E. coli* TolR stabilize the dimeric structure of the protein.

**TABLE 1:** **Analytical ultracentrifugation sedimentation equilibrium data for TolR(36–142)**

Protein concentration	Speed	Calculated molecular mass
μ*m*	*rpm*	*Da*
20	25,000	21,476*^a^*
	30,000	19,921*^a^*
	35,000	19,736*^a^*
200	15,000	20,962*^a^*
	20,000	20,874*^a^*
	25,000	20,335*^a^*
Global fit		20,408*^a^*, 20,433*^b^*

*^a^* This is a self-association model.

*^b^* This is a single ideal species model.

##### Crystal Structure of E. coli TolR(36–142) Reveals a Strand-swapped Dimer

**FIGURE 3.**
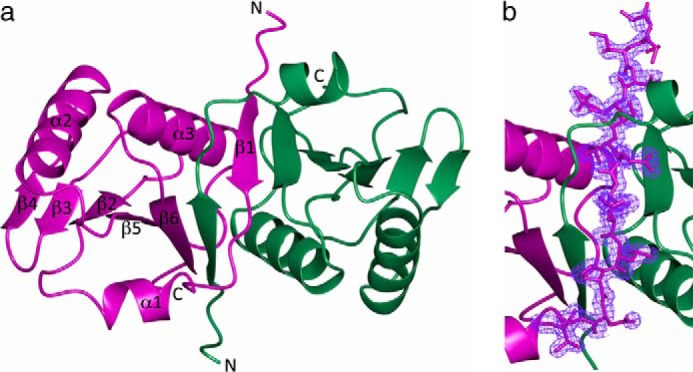
**Crystal structure of *E. coli* TolR(36–142) dimer.** *a*, architecture of the strand-swapped dimer. *b,* N terminus of TolR(36–142) modeled into the electron density map (2*F_o_* − *F_c_* shown at 1σ cutoff).

We determined the crystal structure of *E. coli* TolR(36–142) to determine the molecular basis for the stabilization imparted by its N- and C-terminal regions. The structure was solved by molecular replacement (see under “Experimental Procedures”) to a resolution of 1.7 Å and refined to an *R* factor of 17.5% (*R*_free_ = 21.5%) (Table 2). The final refined structure included residues 37–141, 161 solvent molecules, and 3 sodium ions. The asymmetric unit of *P*6_5_22 space group contained one molecule of TolR(36–142). The monomer is composed of an N-terminal β-strand (β1 residues 41–47) and short α-helix (α1 residues 51–58) followed by a five-stranded mixed β-sheet (β2 residues 64–68; β3 residues 74–78; β4 residues 81–85; β5 residues 106–112, and β6 residues 133–139) sandwiched against two α-helices, α2 (residues 88–102) and α3 (residues 116–130) (Fig. 3). The organization of secondary structure resembles the ribonuclease H-like fold. A DALI search identified representatives from this superfamily, such as pantothenate kinase from *P. aeruginosa* (*Z* score 6.3, 2f9t), *Vibrio cholerae* general secretion pathway protein L (*Z* score 5.7, 2bh1), and *Pseudomonas aeruginosa* Tex RuvX-like domain-like family (*Z* score 5.7, 3bzk). However, ribonuclease H-like proteins are 3-layered and organized as α/β/α, which is not the case for TolR(36–142) where the third α-helical layer is missing.

**TABLE 2:** **Data collection and refinement statistics for TolR(36–142)** Numbers given in parentheses are from the last resolution shell.

**Data collection**	
Space group	*P*6_5_22
Unit cell parameters (Å, °)	*a* = 51.7, *b* = 51.7, *c* = 155.0 *a* = 90.0, *b* = 90.0, *c* = 120.0
Wavelength (Å)	0.920
Resolution range (Å)	38.8–1.7 (1.79−1.70)
Mean *I*/*s*(*I*)	33.7 (5.5)
*R*_sym_*^a^* (linear) (%)	9.7 (83.6)
Redundancy	41.0 (39.3)
No. of observations	588,835
No. of unique reflections	14,354
Completeness (%)	100 (99.8)

**Refinement**	
Resolution range (Å)	38.8–1.7
No. of reflections (working/free)	14,284/716
No. of protein residues	105
No. of sodium	3
No. of water molecules	161
*R*_work_*^b^*/*R*_free_*^c^* (%)	17.5/21.5
*B* average (Å^2^)	19.5
Main chain	20.0
Side chain	26.1
Water molecules	42.1
r.m.s.d. from ideal values	
Bond lengths (Å)	0.007
Bond angles (°)	1.16

*^a^ R*_sym_ = (Σ*_hkl_*Σ*_i_*|*I(hkl*) − 〈*I*(*hkl*)〉)/Σ*_hkl_*Σ*I_i_*(*hkl*), where *I_i_*(*hkl*) is the intensity of the *i*th measurement of reflection (*hkl*), and 〈*I*(*hkl*)〉 is the average intensity.

*^b^ R*_work_ = (Σ*_hkl_*|*F_o_* − *F_c_*|)/Σ*_hkl_F_o_*, where *F_o_* and *F_c_* are the observed and calculated structure factors.

*^c^ R*_free_ is calculated as for *R*_work_ but from a randomly selected subset of the data (5%), which were excluded from the refinement.

Close examination of TolR packing in the crystal revealed the presence of an intertwined dimer where the N-terminal β-strand of one subunit (β1) formed an anti-parallel β-sheet with the C terminus of a symmetry-related molecule (β6) (Fig. 3, *a* and *b*). The TolR(36–142) dimer interface is formed by residues from three regions as follows: the N terminus of the protein (residues 38–53 and 55), which includes β1 and part of α1; residues from the central regions of the protein that include β5 and α3 (residues 106, 108–118, 120–121, 124, and 127–128), and C-terminal β6 (residues 132–141). In total, 12 amino acids were swapped between the two subunits. Intertwining is a common phenomenon in oligomeric proteins. In a recent study by Mackinnon *et al.* (53), over 20% of all surveyed structures in the PDB were found to be intertwined. Of these, the majority (72%) exchange one contiguous segment of polypeptide chain, denoted as S-type, which is the case for TolR(36–142). Four pieces of evidence suggest this strand-swapped structure is not a crystallographic artifact but rather a physiologically relevant structure of the TolR(36–142) dimer. First, the PISA software identified it as a *bona fide* dimer interface. Second, the regions of TolR(36–142) that form the bulk of the intertwined interfaces are precisely those that destabilize the TolR dimer when deleted (Fig. 2), consistent with their importance to the stability of the dimer. Third, the intertwined structure explains why deletion of the C-terminal 9 amino acids in TolR(36–133) is more destabilizing for the dimer than deletion of the N-terminal 28 residues. β6 at the C terminus makes extensive inter- and intramolecular interactions, the former contributing to the hydrophobic core of the dimer, whereas β1 at the N terminus only makes intermolecular contacts. In addition, the C-terminal region of TolR (encompassing β6) has previously been shown to play an important role in stabilizing the TolR dimer *in vivo* (54). Fourth, our strand-swapped dimer structure explains past *in vivo* disulfide bond cross-linking data involving α3 (21), which are otherwise inconsistent with the *H. influenzae* structure (see below). In summary, our data demonstrate the intact periplasmic domain of *E. coli* TolR is an intertwined dimer, a structural state we propose is central to the mechanism by which TolR functions as the Tol-Pal stator.

##### Structural Comparison of E. coli and H. influenzae TolR, Same Monomer, Different Dimer

**FIGURE 4.**
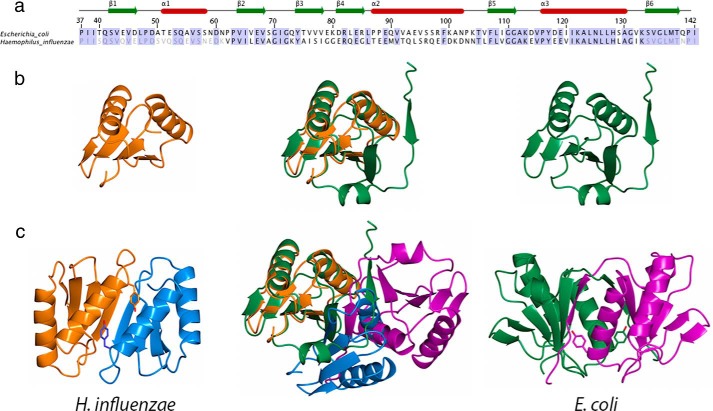
**Comparison of the sequences and structures of *H. influenzae* and *E. coli* TolR periplasmic domains.** *a*, sequence alignment of the periplasmic domains of *E. coli* and *H. influenzae* overlaid with the secondary structure for the *E. coli* protein. *b,* structural comparison and superposition of truncated *H. influenzae* TolR (TolR(60–133) in *orange*) and intact *E. coli* TolR (TolR(36–142) in *green*). *c,* comparison of the TolR dimer structures from *H. influenzae* and *E. coli* highlighting the position of Tyr-117 from helix α3, which is involved in stabilizing the interface of both structural forms. An overlay of both dimer structures, superimposed on one subunit, emphasizes the change in oligomeric structure.

The high sequence identity of the *E. coli* and *H. influenzae* TolR proteins suggests their periplasmic domains should have a similar if not identical fold. Indeed, our structure for *E. coli* TolR(36–142) was solved, in part, using the *H. influenzae* NMR solution structure as a search model in molecular replacement. Unsurprisingly then, *E. coli* and *H. influenzae* TolR monomers share a high degree of structural similarity (r.m.s.d. 1.41 Å^2^ for 74 Cα atoms) for those regions of the sequence they share; residues 60–133 have *E. coli* numbering (Fig. 4, *a* and *b*). The additional sequences in *E. coli* TolR(36–142) include β1/α1 at the N terminus and β6 at the C terminus. The presence of these additional 37 residues results in a radically different quaternary structure for the TolR dimer; rotations of ∼180° along two axes are required to transform *E. coli* to the *H. influenzae* dimer (Fig. 4*c*). Below we summarize the principal differences between the two dimer structures, where we refer to the *H. influenzae* structure as a “truncated TolR” in which β1/α1 and β6 are missing, and we suggest they likely represent different functional states of TolR in the bacterial periplasm.

The most striking difference between the two structures is that truncated TolR is not an intertwined dimer. The β-strands of truncated TolR (β2-β5, *E. coli* numbering) form a contiguous β-sheet structure across the interface giving the structure its characteristic curved architecture. Intertwining in TolR(36–142) obliterates the large groove that runs between the subunits in truncated TolR. Moreover, the additional sequences within TolR(36–142) change the overall symmetry of the dimer from an anti-parallel arrangement of subunits in truncated TolR to parallel in TolR(36–142). These differences in orientation are epitomized by the interactions of helix α3, which is involved in forming the dimer interface in both structures. In truncated TolR, helix α3 is in an anti-parallel arrangement, and its residues, such as Tyr-117 at the C-terminal end of the helix (Fig. 4*c*) form hydrophobic contacts (Ile-121, Leu-124, Asn-125, and His-128) and a hydrogen bond (Ile-121) with opposing α3 residues. In contrast, the α3 helices of TolR(36–142) are parallel and tilted by 60°, with far fewer contacts (Ile-120) between residues of the opposing helix. The interactions of Tyr-117 are also different; its side chain is buried deep in the opposing monomer in TolR(36–142) and forms a hydrogen bond with β5 Ile0109 that is not formed in truncated TolR. Not only are the interactions of Tyr-117 different between the two TolR structures, but the Cα distance between opposing Tyr-117 side chains within the dimer is also different (Fig. 4*c*); in truncated TolR, the Tyr-117–Tyr-117 distance is 15.2 Å, and this distance is markedly shorter in TolR(36–142) (5.6 Å), which is close enough to form an intersubunit disulfide bond, as reported by Goemaere *et al.* (21). The number of residues involved and the interactions formed at the TolR dimer interface are also very different in the two structures. In truncated TolR, ∼1700 Å^2^ of accessible surface area is buried at the dimer interface involving 24 residues that form mostly hydrophobic interactions but also six interfacial hydrogen bonds (Table 3). In contrast, >2200 Å^2^ of accessible surface area is buried at the dimer interface of TolR(36–142), involving 44 residues from each monomer stabilized by 34 direct hydrogen bonds and 2 salt bridges. The significantly greater number of stabilizing interactions in TolR(36–142) therefore explains its greater stability relative to truncated TolR. The majority of residues involved in stabilizing the truncated TolR dimer (β5 and α3) also stabilize TolR(36–142), but they form completely different interactions, as exemplified by Tyr-117. The greater number of stabilizing interactions within the TolR(36–142) dimer implies the intertwined N- and C-terminal sequences block formation of the anti-parallel arrangement of subunits in preference for a parallel arrangement. Conversely, this implies that in order for the truncated dimer structure to form the N- and C-terminal linker, sequences must be removed.

**TABLE 3:** **Interface comparison statistics of *E. coli* Tol(36–142) (this work) and truncated *H. influenzae* TolR (24)**

	*E. coli* TolR(36–142)			*H. influenzae* TolR(62–133)*^a^*		
	Chain 1	Chain 2	Distance	Chain 1	Chain 2	Distance
			Å			Å
1	Gln-41(N)	Thr-139(OG1)	3.03	Val-109(O)	Val-109(N)	2.80
2	Val-43(N)	Leu-137(O)	3.02	Lys-133(NZ)	Val-115(O)	2.93
3	Val-45(N)	Val-135(O)	2.91	Tyr-117(OH)	Ile-121(O)	2.97
4	Asp-46(N)	Asp-46(O)	2.86	Val-109(O)	Val-109(N)	2.79
5	Leu-47(N)	Lys-133(O)	2.81	Val-115(O)	Lys-133(NZ)	2.93
6	Tyr-117(N)	Lys-133(O)	3.15	Ile-121(O)	Tyr-117(OH)	2.94
7	Tyr-117(N)	Val-115(O)	2.79			
8	Tyr-117(OH)	Ile-109(O)	2.76			
9	Asp-118(N)	Lys-113(O)	3.05			
10	His-128(NE2)	Leu-47(O)	3.40			
11	Ser-134(OG)	Asp-46(OD1)	2.94			
12	Val-135(N)	Glu-44(OE2)	3.77			
13	Val-135(N)	Val-45(O)	2.89			
14	Leu-137(N)	Val-43(O)	2.82			
15	Met-138(N)	Tyr-117(OH)	3.24			
16	Thr-139(N)	Gln-41(O)	3.11			
17	Thr-139(OG1)	Gln-41(O)	3.59			
18	Thr-139(OG1)	Gln-41(N)	3.03			
19	Leu-137(O)	Val-43(N)	3.02			
20	Val-135(O)	Val-45(N)	2.91			
21	Asp-46(O)	Asp-46(N)	2.86			
22	Lys-133(O)	Leu-47(N)	2.81			
23	Val-115(O)	Tyr-117(N)	2.79			
24	Lys-113(O)	Tyr-117(N)	3.15			
25	Ile-109(O)	Tyr-117(OH)	2.76			
26	Lys-113(O)	Asp-118(N)	3.05			
27	Leu-47(O)	His-128(NE2)	3.40			
28	Asp-46(OD1)	Ser-134(OG)	2.94			
29	Val-45(O)	Val-135(N)	2.89			
30	Glu-44(OE2)	Val-135(N)	3.77			
31	Val-43(O)	Leu-137(N)	2.82			
32	Tyr-117(OH)	Met-138(N)	3.24			
33	Gln-41(O)	Thr-139(N)	3.11			
34	Gln-41(O)	Thr-139(OG1)	3.59			
35*^a^*	His-128(NE2)	Asp-49(OD1)	3.37			
36*^a^*	Asp-49(OD1)	His-128(NE2)	3.37			

*^a^* Salt bridges are shown.

*^b^ E. coli* numbering of residues is used.

##### Cryptic Peptidoglycan Binding Activity Is Observed for Truncated TolR

**FIGURE 5.**
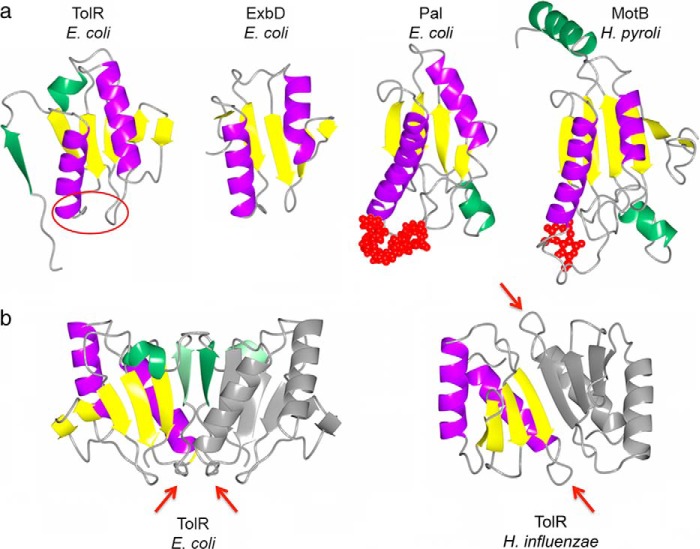
**Structural comparison of bacterial OmpA-like PG binding domains.** *a,* structures *of E. coli* TolR(36–142), *E. coli* ExbD (PDB code 2pfu), *E. coli* Pal (PDB code1oap; UDP-*N*-acetylmuramoyl-l-alanyl-d-glutamyl-*meso*-2,6-diaminopimeloyl-d-alanyl-d-alanine PDB code 2aiz), and *H. pylori* MotB (PDB code 3s0y; *N*-acetylmuramic acid PDB code 3cyq) share similar structural features. A central 4- or 5-stranded β-sheet (shown in *yellow*) is sandwiched against two α-helices (shown in *purple*). Structures are decorated with additional unique secondary structure elements (shown in *green*). Ligands are shown as *red spheres* for Pal and MotB, whereas the putative *E. coli* TolR(36–142) PG-binding site is indicated with a *red circle. b,* potential PG-binding sites in the *E. coli* TolR(36–142) and *H. influenzae* TolR(62–133) dimers are indicated with *red arrows*. In *H. influenzae* TolR(62–133), the binding sites lie on opposite sides of the dimer, as for MotB. By analogy with the modeling prediction of PG binding to MotB (55), the stem peptide would sit between the β-sheet within the deep intersubunit groove. In *E. coli* TolR(36–142), these sites are not only overlapping, which would block PG binding, but also face the inner membrane.

**FIGURE 6.**
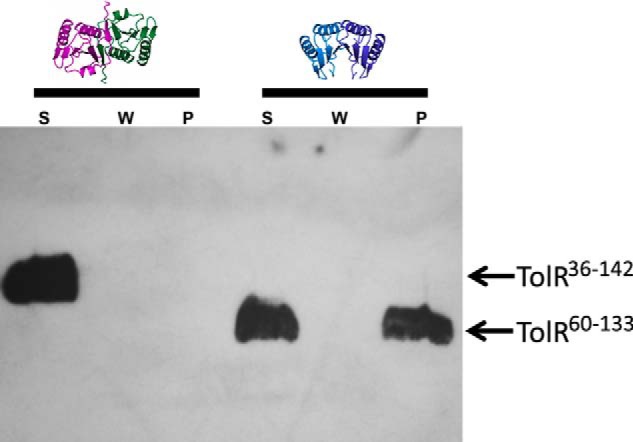
**TolR binds PG only after its N- and C-terminal strand-swapped sequences have been deleted.** Western blot of TolR(36–142) (*left-hand three lanes*) and TolR(60–133) (*right-hand three lanes*) using anti-His antibody detecting a C-terminal histidine tag. The three lanes for each protein sample represent the supernatant following incubation with *E. coli* sacculi (*S*), the supernatant after three wash steps (*W*), and the pellet fraction (*P*). Only truncated TolR (TolR(60–133)) pellets with sacculi suggesting the additional sequences in TolR(36–142) prevent binding of the protein to PG.

Although TolR shares limited sequence identity with ExbD from the Ton system, they have similar structures (r.m.s.d. 3.2 Å^2^ for 61 Cα atoms). They are OmpA-like proteins, a diverse family of proteins implicated in binding PG (Fig. 5). OmpA-like proteins include OM-attached PG-associated lipoprotein (Pal; r.m.s.d. 3.7 Å^2^ for 63 Cα atoms) from the Tol-Pal system and MotB from the bacterial flagellum (r.m.s.d. 3.1 Å^2^ for 58 Cα atoms). Both Pal and MotB have been shown, by NMR and crystallography, respectively, to bind fragments of PG (18, 55). A model for PG binding has been proposed for MotB in which two glycan chains bind at the ends of the β-sheet, and the peptide cross-bridge connecting the glycan chains binds within the canyon at the dimer interface (55). We therefore investigated whether truncated and full-length *E. coli* TolR bound PG in a pulldown assay using intact *E. coli* sacculi and His-tagged TolR (see “Experimental Procedures”). Truncated TolR (TolR(60–133)) bound PG but TolR(36–142) did not (Fig. 6). We conclude that the intertwined N- and C-terminal sequences of the intact periplasmic domain of TolR block its ability to bind PG by reconfiguring the dimer interface.

##### Molecular Dynamics Simulations Suggest TolR(36–142) Is a Stable Dimer at the Surface of the Phospholipid Bilayer

**FIGURE 7.**
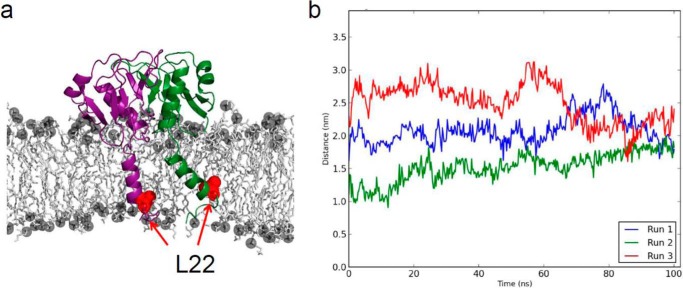
**MD simulations of intact *E. coli* TM-TolR(36–142) in an anionic phospholipid bilayer shows the dimer to be stable at the membrane surface.** *a*, snapshot of the MD simulation showing the relative positions of Leu-22 within the membrane. *b, graph* showing the convergence of the Leu-22–Leu-22 distance to 20 Å during the 100-ns MD simulation of TM-TolR(36–142), which is too far to form a disulfide bond as suggested previously by Zhang *et al.* (22). See text for details.

Parsons *et al.* (24) have proposed previously that truncated TolR represents the structure of the dimer close to the membrane surface. However, this structure is missing the 58 N-terminal residues, including the 25 residues that link the transmembrane helix to the periplasmic domain. The *E. coli* structure reported here contains the entire periplasmic domain, with just a single residue missing from the electron density. Hence, it is more likely TolR(36–142) represents the structure of the TolR dimer close to the surface of the inner membrane; truncated TolR represents an alternative conformation away from the membrane (see below). To evaluate the stability of the membrane-bound form of the TolR(36–142) dimer, we modeled the protein with the transmembrane region (residues 15–36, TM-TolR(36–142)) and conducted molecular dynamics simulations (100 ns duration) of the protein embedded in a model of the *E. coli* inner membrane (4:1, phosphatidylethanolamine/phosphatidylglycerol) (see under “Experimental Procedures”). Although the MD simulations were missing the additional transmembrane helices of TolA and TolQ from the Tol-Pal complex (the structures of which are unknown), they nonetheless highlighted two important aspects of the TolR dimer. First, the TM-TolR(36–142) dimer was stable in the bilayer (Fig. 7*a*), with little evidence of disruption of the dimer interface even though TolR has a negatively charged surface proximal to the membrane (data not shown). This appears to be allowed due to clustering of the zwitterionic head groups of the phosphatidylethanolamine lipids around the negative charge and through interactions with sodium ions. The critical aspartate (Asp-23) within the TM helix of TolR, which is the likely site of protonation for pmf coupling of the Tol-Pal complex (20), was bound by a single sodium ion during the simulation, indicating it is accessible to ions despite being within the bilayer. Second, the architecture of the intertwined dimer positions the TM helices of TolR far apart, and they remain separated throughout the simulation (Fig. 7*b*). The lack of contact between the TM helices of TolR contrasts with what has been proposed previously based on *in vivo* cysteine-disulfide cross-linking studies, where a disulfide bond readily formed between L22C residues within the TM helix (22). We propose that one of the functions of the TM helices of TolA and TolQ, in conjunction with the pmf, is to bring the TM helices of the TolR dimer closer together.

## Discussion

### 

#### 

##### TolR Undergoes a Structural Transition Similar to That of MotB to Contact the Bacterial Cell Wall

**FIGURE 8.**
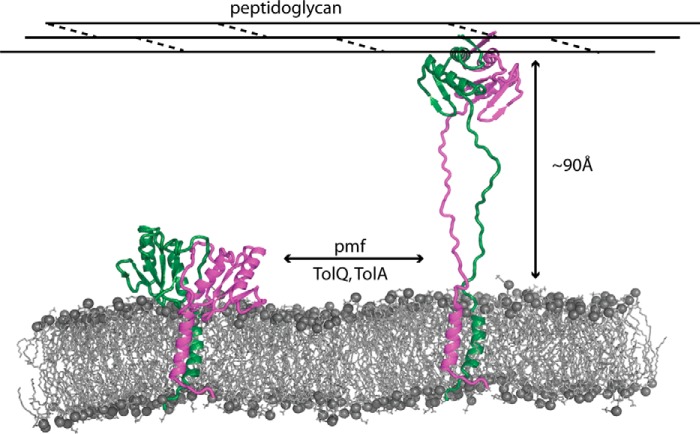
**Postulated structural transition of the TolR dimer between the inner membrane, represented by *E. coli* TolR(36–142) from this work, and PG layer, represented by truncated TolR from *H. influenzae* (24).** The figure illustrates the type of structural transition TolR likely undergoes to cycle between the two compartments of the cell envelope, fueled by the pmf and the interactions of TolQ and TolA in the inner membrane. A similar model has been proposed previously for the bacterial flagellar stator protein MotB (58, 59). We propose the structure of TolR close to the membrane represents the closed state of the proton pore and that of the extended structure represents the open state of the pore.

An essential feature of MotA/MotB stator function is cycling of the periplasmic domain of the MotB dimer between the IM and PG layer, which are separated by ∼90–100 Å in the bacterial periplasm. Structures of MotB from *Salmonella typhimurium* and *Helicobacter pylori* have provided clues as to the structural transition that underpins this movement. The intact periplasmic domain of MotB is a domain-swapped dimer in which the folded N-terminal linker of one subunit interacts with the C-terminal α-helix of the other subunit. These interactions serve two purposes. First, they retain the dimer at the IM as there is insufficient sequence at the N terminus to allow the protein to reach the PG layer. Second, they occlude residues required for PG binding. The structure for the intact periplasmic domain of *E. coli* TolR illustrates a similar mechanism albeit differing in detail. Domain swapping in TolR is mediated by β-strands of the protein at its N and C terminus rather than α-helices, but the end result is nevertheless the same, the inability of the intact periplasmic domain of TolR to bind PG (Fig. 6). MotB/TolR are also thought to “plug” the proton pore of their respective complexes, which is consistent with their periplasmic domains lying close the membrane surface. In the case of TolR, the C-terminal 27 amino acids of the domain have been shown to be important in maintaining the “closed” state of the stator pore, although these previous studies inferred the C-terminal 27 amino acids interacted with the membrane in a pmf-dependent fashion (21). In fact, it is clear from the structure of TolR(36–142) that the C-terminal 27 amino acids of the domain (which includes the C-terminal β3) are critical for stabilizing the intertwined dimer state. Hence, this form of the stator protein likely represents the closed state of the pore, close to the membrane surface. *In vivo* disulfide bond engineering data also support such a role. As described above, Tyr-117 is only close enough to form a disulfide bond when TolR is strand-swapped. Indeed, this disulfide was the only one to form spontaneously *in vivo* of the 27 Cys mutations generated in the C-terminal regions of TolR (21). It follows that if TolR(36–142) is the closed state of the TolQR proton-conducting pore, a Y117C disulfide within the dimer should (i) inactivate the Tol-Pal function and (ii) form regardless of whether the proton conductance channel is disrupted. Goemaere *et al.* (21) reported both effects for TolR Tyr117Cys. Indeed, Goemaere *et al.* (21) commented that the Y117C disulfide trapped the dimer in the “nonenergized form of the complex,” which is consistent with the strand-swapped dimer being the “closed state” of the Tol-Pal stator *in vivo*. Truncated forms of MotB and TolR missing the strand-swapped sequences restructure the dimer and expose PG binding regions in the protein. Hence, the periplasmic domains of both MotB and TolR must undergo large scale structural remodeling in order for their strand-swapped regions to dissociate from the main body of the protein and expose their PG-binding sites. In the case of TolR, we suggest that the deep groove seen in the structure of truncated TolR is the PG-binding site, as has been suggested for MotB (55). Such restructuring would undoubtedly require force (34 hydrogen bonds must be broken in the intertwined TolR dimer) with this force delivered by the pmf in association with the partner proteins, TolA and TolQ, respectively. Consistent with this interpretation, previous cysteine accessibility studies have demonstrated that the C terminus of TolR undergoes structural rearrangement in response to the pmf (21). Because this includes the C-terminal β3-strand, which as our SEC-MALLS data show is the most destabilizing for the dimer when deleted, dissociation of this strand most likely initiates separation of the N-terminal linker sequence with which it is strand-swapped. Once dissociated, the linker sequence of TolR (and MotB) is of sufficient length (>25 amino acids) to allow the OmpA-like domain to contact the PG layer ∼90 Å away (Fig. 8). This form of the stator likely represents the open pore of the stator complex through which protons flow.

##### Rotation and Closer Juxtaposition of the Transmembrane Helices of the Tol-Pal Complex Likely Drive the TolR Structural Transition in the Periplasm

Previous work has shown that interactions between the TM helices of TolQ and TolR change in response to the pmf and are mediated by critical charged/polar residues within the membrane (*e.g.* TolR Asp-23) (20–23). Equivalent residues are also present in the MotA-MotB complex and indeed other pmf-coupled machines in the periplasm. The movement of protons through the TolQ/TolR pore are thought to cause the TM helices of TolR to rotate relative to those of TolQ. A similar rotation of TM helices has been proposed for the MotA-MotB complex (56, 57). However, our MD simulations suggest the TM helices of TolR may only interact when the periplasmic domain is not strand-swapped. This implies that an additional function of the TM helices of TolQ and TolA within the Tol-Pal complex might be to bring the TolR TM helices closer together. We suggest that it is the combination of rotation and closer juxtaposition of the TM helices of TolR that provide the lateral force needed to remodel the strand-swapped dimer at the membrane surface, simultaneously exposing the PG-binding residues and allowing the domain to extend through the periplasm (Fig. 8).

In both TolR and MotB, multiple hydrogen bonds within intertwined regions of their dimer structures have to be broken for their periplasmic domains to bind PG. Yet the mode of strand swapping is specific to each dimer. From this, we infer that the force generated by the movement of protons through the MotAB and TolQR stators is most effective at eliciting the requisite structural reorganization of the MotB/TolR dimer when the periplasmic domain is strand-swapped regardless of the type of structures that are swapped. We anticipate similar mechanisms will be found in other bacterial motor stator proteins.

## Author Contributions

C. K. conceived and coordinated the study, contributed to the interpretation of the data. and wrote the paper. J. A. W. purified proteins, undertook solution experiments, solved the crystal structure of TolR, and interpreted the structure. E. C. and P. J. S. conducted MD simulations of full-length TolR. R. K. constructed all expressing plasmids and purified proteins. G. P. conducted the sacculi binding experiments on TolR constructs. J. T. S. H. and C. V. R. conducted native state mass spectrometry experiments. All authors analyzed the results and approved the final version of the manuscript.

## Footnotes

4 The abbreviations used are:

OM: outer membrane
IM: inner membrane
PG: peptidoglycan
pmf: proton-motive force
TM: transmembrane
SEC-MALLS: size-exclusion chromatography multiangle laser light scattering
PDB: Protein Data Bank
LDAO: lauryldimethylamine oxide
r.m.s.d.: root mean square deviation
MD: molecular dynamics.
